# Supplementary material for: LSTM-based estimation of lithium-ion battery SOH using data characteristics and spatio-temporal attention
Source: PLoS One. 2024 Dec 26;19(12):e0312856. doi: 10.1371/journal.pone.0312856 (PMC11671002; doi:10.1371/journal.pone.0312856)
Supplement: Attachment — Submitted filename: Response to Reviewers.doc [file pone.0312856.s001.doc]

Dear Editors and Reviewers:

We have made every effort to enhance the manuscript and have incorporated the suggested changes. These revisions do not affect the content or framework of the paper. We are grateful for the diligent work of the Editors and Reviewers, and we have addressed each reviewer's comments individually. We hope that these corrections meet with your approval.

**Reviewer #1**

This paper tries to propose a novel battery SOH estimation strategy based on combination of LSTM model and Spatio-Temporal Attention method. However, the authors fail to clearly demonstrate how the proposed strategy working and why it is better than others. Besides, there are lots of grammar, writing and organizing deficiencies of this manuscript.

**Response:**

Thank you for your comments and suggestions for our manuscript. We have carefully revised the manuscript to make some issues clearer. Additionally, we have invited one of our colleagues to help enhance the manuscript's language.The detailed responses to your comments are given as follows.

**Comment 1**

The writing and organizing of this manuscript are far from satisfactory and professional. For example:

a) The name of authors in the information table.

b) The figure quality in this manuscript is less appreciate, including font, size, capitalize, and unit.

c) The table in this paper is also low quality, including font, header name, organization, key information highlight, and so on.

d) What is the meaning of ‘L’ in the vector parameters?

e) There are serious formatting issues with the formula and the related explanation.

f) Inconsistent capitalization in Section titles.

**Response:**

Thanks for your comments. The modifications to the above six points have been completed, and the specific details are as follows.

a) In the information table, the initials of the author ‘jingyun xu’ were not capitalized, and they have now been corrected to ‘Jingyun Xu’.

b) The images in the manuscript have been enhanced, and the content has been revised accordingly.

c) The fonts, title names, and key information have been reformatted, improving the quality of the tables.

d) 'L' represents the long-term trend data synchronized at time k, obtained by averaging the capacity values over the past M time steps. This reflects the overall degradation trend of the battery capacity and is useful for capturing the smooth changes in capacity over time, effectively mitigating the impact of short-term fluctuations on prediction results.

e) A thorough review of the formulas and explanations in the entire text has been completed, and errors in content and formatting have been corrected.

f) The size of the chapter headings has been revised.

**Comment 2**

The NASA datasets have been widely used in numerous articles and are outdated. The authors should examine the proposed method based on other recent datasets with different battery types.

**Response:**

Thanks for your comments. We understand your concerns regarding the diversity of the dataset and are happy to provide further clarification. First, the NASA lithium-ion battery dataset is still widely used in the field of battery State of Health (SOH) prediction due to its high data quality, strict testing conditions, and broad coverage. The NASA dataset not only provides detailed battery cycle data but also includes degradation processes of various battery types, making it a standard dataset in the industry. Although the dataset was generated some time ago, it remains highly authoritative due to the rigor of its experimental design and the high consistency of the data. It has been used by many research institutions and scholars to validate the effectiveness of new methods. Additionally, we chose this well-recognized and widely-used dataset to compare the performance of different algorithms. By using the NASA battery dataset, our model can be benchmarked against existing SOH prediction algorithms. However, we fully understand the reviewer's suggestion to extend the dataset. In our future work, we plan to introduce a lithium battery degradation dataset provided by our industry partner, Tianneng Battery Group Co., Ltd., to further validate the model.

**Comment 3**

Four health factors are very important in this paper but the authors ignore to describe them clearly. Meanwhile, these factors are all related with charging time, which is possible to be redundant.

**Response:**

Thank you for your comments. We realize that the description of the four health factors in the original manuscript may not have been sufficiently clear, so we have provided a more detailed explanation below and incorporated it into the paper. (F1 represents the battery performance in the 3.8V-3.9V range, primarily reflecting capacity changes in the initial phase; F2 represents the 3.9V-4.0V range, reflecting the battery's mid-term health state; F3 represents the 4.0V-4.1V range, tracking the battery's performance as it approaches full charge; F4 represents the 4.1V-4.2V range, describing the battery's behavior under high voltage conditions.) The four health factors in this paper represent the battery's performance across different voltage ranges. Although they are related to charging time, they primarily reflect the battery's capacity changes at different voltage levels, and there is no issue of redundancy. We will further clarify the physical meaning and significance of each health factor in the paper to prevent any potential misunderstanding by the readers.

**Original:**

This paper selects three sets of data, labeled B5, B6, and B7, as the subjects for health factor extraction. Under the CC charging mode, the time required for the battery voltage to move through four stages: 3.8V-3.9V (F1), 3.9V-4.0V (F2), 4.0V-4.1V (F3), and 4.1V-4.2V (F4) is recorded^[27]^.

**Revised:**

This paper selects three sets of data, labeled B5, B6, and B7, as the subjects for health factor extraction. Under the CC charging mode, the time required for the battery voltage to move through four stages: 3.8V-3.9V (F1), 3.9V-4.0V (F2), 4.0V-4.1V (F3), and 4.1V-4.2V (F4) is recorded[27]. Among them,F1 represents the battery performance in the 3.8V-3.9V range, primarily reflecting capacity changes in the initial phase; F2 represents the 3.9V-4.0V range, reflecting the battery's mid-term health state; F3 represents the 4.0V-4.1V range, tracking the battery's performance as it approaches full charge; F4 represents the 4.1V-4.2V range, describing the battery's behavior under high voltage conditions.

**Comment 4**

The conclusion of correlation analysis is unclear. Which factor should be selected and used? Why the authors emphasize the correlation between capacity and factors? In fact, there are lots of discussion without conclusive result.

**Response:**

Thanks for your comments. According to the results of the correlation analysis, F1, F2, and F3 show a strong correlation with battery capacity, with correlation coefficients exceeding 0.9 across different voltage ranges. However, during the actual experiments, the predictive performance of the F1 factor did not meet expectations. Experimental results indicate that when F1 is used, the model's prediction accuracy is relatively low, with significant errors in certain voltage ranges. In contrast, the F2 and F3 factors exhibited higher stability and accuracy in the experiments. Based on these experimental results, we ultimately selected F2 and F3 as the primary health factors. This aspect was indeed insufficiently explained in the original paper, and we have now added the necessary content.

**Revised:**

Since users typically charge the battery when there is still remaining capacity, the health factor F1 may have missing data. Subsequent experimental validation shows that F2 and F3 perform best as health factors, and therefore, F2 and F3 are selected for further research in this paper.

**Comment 5**

How to divide the degradation data into trend data and short-term data? What is the definition of the trend and short-term data?

**Response:**

Thanks for your comments. Trend data reflects the long-term degradation behavior of the battery, indicating that there is little change in multiple sampling points around time k, which is manifested as relatively smooth sections on the degradation curve. In this paper, this is considered a trend feature. Short-term data represents short-term fluctuations or anomalies, such as capacity regeneration points, which appear as "peaks" on the degradation curve. In this paper, these are considered short-term features.

**Comment 6**

It is hard to fully understand the Fig.4 without explicit explanation.

**Response:**

Thanks for your comments. Fig.4 is an overall algorithm structure diagram. The original flowchart lacked annotations, making it difficult to follow. The flowchart has now been re-optimized, and textual descriptions have been added.

**Original:**

**Revised:**

Block I is a feature optimization module based on the spatial attention mechanism. This module mainly addresses the issue of feature selection by combining the health factors F2, F3, and *Q* to form a three-dimensional vector, and using the attention mechanism to calculate weights, enabling the model to better capture the patterns of battery capacity changes over time.Block II is a feature optimization module based on the temporal attention mechanism. This module focuses on dynamic changes in the time series, paying special attention to key time points. It effectively addresses prediction errors caused by fluctuations in time features during battery capacity prediction, further improving the model's stability and prediction accuracy.

**Comment 7**

Section Ⅲ is the core of this paper but describe confusedly. Thus, the readers can hardly understand the contribution and innovation of this work.

**Response:**

Thanks for your comments. Based on your feedback, we have refined the key innovations at the beginning of Section 3 and provided new explanations in conjunction with the algorithm flowchart.

**Original:**

To address the trend-based nonlinearity in degradation sequences, which initially show a gradual decline followed by a rapid decrease, the degradation data are divided into trend data and short-term data. Then, considering the significant dynamic changes among samples, especially in terms of capacity regeneration phenomena, a spatio-temporal attention mechanism for short-term data is designed to extract short-term spatio-temporal features^[28]^. Subsequently, an LSTM model is established using trend data, short-term spatio-temporal features, estimated capacity, and actual capacity as inputs to estimate subsequent capacity. The overall structure of the algorithm is shown in Figure 4.

**Revised:**

To address the trend-like nonlinearity of degradation sequences, which is initially gradual and then accelerates, the degradation data is divided into trend data and short-term data. Then, to handle the significant dynamic variations between samples, particularly in the case of capacity regeneration, a spatio-temporal attention mechanism is designed for short-term data to extract short-term spatio-temporal features [28]. An LSTM model is then constructed using trend data, short-term spatio-temporal features, estimated capacity, and actual capacity as inputs to estimate future capacity. The overall structure of the algorithm is shown in Fig 4. Block I is a feature optimization module based on the spatial attention mechanism. This module mainly addresses the issue of feature selection by combining the health factors F2, F3, and *Q* to form a three-dimensional vector, and using the attention mechanism to calculate weights, enabling the model to better capture the patterns of battery capacity changes over time.Block II is a feature optimization module based on the temporal attention mechanism. This module focuses on dynamic changes in the time series, paying special attention to key time points. It effectively addresses prediction errors caused by fluctuations in time features during battery capacity prediction, further improving the model's stability and prediction accuracy.

**Comment 8**

The authors could investigate the public paper for better writing and organizing. For example:

a) Battery SOH estimation method based on gradual decreasing current, double correlation analysis and GRU

b) Performance simulation method and state of health estimation for lithium-ion batteries based on aging-effect coupling model

c) Accurate state of health estimation of battery system based on multi-stage constant current charging and behavior analysis in real-world electric vehicles

**Response:**

Thanks for your comments. We have carefully reviewed and studied the paper you mentioned and have added it to the references.

1. Chaolong Zhang, Laijin Luo, Zhong Yang, Shaishai Zhao, Yigang He, Xiao Wang, et al.Battery SOH estimation method based on gradual decreasing current, double correlation analysis and GRU,Green Energy and Intelligent Transportation,Volume 2, Issue 5,2023,100108,ISSN 2773-1537,https://doi.org/10.1016/j.geits.2023.100108.
2. Deyu Fang, Wentao Wu, Junfu Li, Weizhe Yuan, Tao Liu, Changsong Dai, et al.Performance simulation method and state of health estimation for lithium-ion batteries based on aging-effect coupling model,Green Energy and Intelligent Transportation,Volume 2, Issue 3,2023,100082,ISSN 2773-1537,https://doi.org/10.1016/j.geits.2023.100082.
3. C. She, Y. Shen, G. Bin, J. Hong and Y. Peng, "Accurate State of Health Estimation of Battery System based on Multi-stage Constant Current Charging and Behavior Analysis in Real-world Electric Vehicles," in IEEE Transactions on Transportation Electrification, doi: 10.1109/TTE.2024.3386831.

**Reviewer #2**

This paper introduces an advanced lithium-ion battery SOH estimation method using LSTM network, which combines trend and non-trend features with a spatiotemporal attention mechanism. The method effectively addresses trend-based nonlinearities and significant dynamic changes in SOH degradation sequences.

**Response:**

Thank you for your comments and suggestions regarding our manuscript. We have carefully revised the manuscript to improve clarity. The detailed responses to your comments are as follows.

**Comment 1**

In section 2, the five different discharge rates and intervals were not visualized, it is recommended to add some details about it.

**Response:**

Thank you for your comments. To better explain the relationship between different discharge rates and intervals, we have made some modifications to the original figure. The figure shows the voltage curves under different discharge cycles (40 cycles, 70 cycles, 100 cycles, 130 cycles, and 160 cycles). As the number of discharge cycles increases, the battery's charging curve gradually shifts to the right, indicating that as the battery ages, the charging time increases, and the rate of voltage rise slows down. The inset in the figure further enlarges the high-voltage region of the charging process, providing a detailed view of the subtle voltage changes between different discharge cycles. With this image, we aim to clearly demonstrate the performance differences of the battery at different discharge rates and intervals, helping readers to better understand the characteristics of the battery at different stages of degradation.

**Original:**

**Revised:**

**Comment 2**

The reviewer wants to know why the author choose B5,B6,B7 among the NASA batteries datasets as the subjects for health factor extraction.

**Response:**

Thank you for your comments. We selected batteries B5, B6, and B7 as the subjects of our study for the following reasons: First, they exhibit similar aging patterns in specific usage scenarios, ensuring consistency. Choosing these batteries ensures that the extracted health factors are stable and reproducible. Second, the B5, B6, and B7 batteries display clear degradation characteristics over time, which can be effectively combined with the spatio-temporal attention mechanism to improve the model's ability to estimate the battery's state of health.

**Comment 3**

On page 7, line 11, “For the batteries B5, B7, the F1 health factor shows the strongest correlation with the capacity degradation value.” it is illustrated in table1 that it is the health factor F4 of B7 shows the strongest correlation.

**Response:**

Thanks for your comments.According to the results of the correlation analysis, F1, F2, and F3 show a strong correlation with battery capacity, with correlation coefficients exceeding 0.9 across different voltage ranges. However, during the actual experiments, the predictive performance of the F1 factor did not meet expectations. Experimental results indicate that when F1 is used, the model's prediction accuracy is relatively low, with significant errors in certain voltage ranges. In contrast, the F2 and F3 factors exhibited higher stability and accuracy in the experiments. Based on these experimental results, we ultimately selected F2 and F3 as the primary health factors. This aspect was indeed insufficiently explained in the original paper, and we have now added the necessary content.

**Revised:**

Since users typically charge the battery when there is still remaining capacity, the health factor F1 may have missing data. Subsequent experimental validation shows that F2 and F3 perform best as health factors, and therefore, F2 and F3 are selected for further research in this paper.

**Comment 4**

The reviewer would like to know that why F4 health factor of B6 shows a big difference comparing with other factors.

**Response:**

Thanks for your comments.The B6 battery may be in a more severe state of aging, and its performance instability increases in the high voltage range (4.1V-4.2V). We observed that for the B6 battery, the F4 health factor behaves abnormally in this voltage range, primarily due to the accelerated chemical reaction rate in the high voltage region, which shortens the charging time and reduces the effective charge input. This directly affects the calculation of the F4 health factor, causing it to exhibit significant volatility and instability. Therefore, in this paper, we chose not to include F4 as an input feature to ensure the stability of the model and improve prediction accuracy.

**Comment 5**

The reviewer wonders that what are the specific meanings and advantages of the performance evaluation metrics (such as RMSE) the author chose respectively in this experiment.

**Response:**

Thanks for your comments. RMSE is the square root of the ratio of the squared deviation between the predicted value and the true value to the number of observations nnn. MAE represents the mean absolute error between the predicted and observed values. In this paper, RMSE and MAE are used to calculate the error between the estimated SOH value and the actual SOH value at time kkk. Lithium-ion battery SOH estimation needs to account for both long-term degradation trends and short-term fluctuations. RMSE is chosen to ensure that the model is sensitive to large errors, preventing excessive prediction deviations. MAE is selected to measure the impact of short-term fluctuations on overall error, preventing extreme errors from overly influencing the evaluation results.

**Comment 6**

On page 17, line 22, “Using features from the current cycle, the model has a good ability to generalize to unseen data.” there are not adequate descriptions that supporting this view, it is recommended to add some further explanations.

**Response:**

Thanks for your comments.The analysis of Figures 9 and 10 in the original text was somewhat unclear and failed to accurately summarize the experimental conclusions. We have now made a comprehensive revision to this section.

**Original:**

1)For the feature selection method, using health factors from the current cycle significantly improves the prediction accuracy, and the selected health factors are highly effective. The features from the current cycle are more representative of the current battery state and more relevant for the current cycle SOH estimation. In contrast, the features from the previous cycle are less representative for predicting the current cycle SOH. This is consistent with the hypothesis that using features from the current cycle, after three iterations of feature optimization, can effectively improve the predictive accuracy.

2)When the battery degradation reached 50% and 70% SOH, respectively, the method for feature selection significantly improved the SOH prediction accuracy. Using features from the current cycle, the model has a good ability to generalize to unseen data. In the 50% and 70% SOH degradation stages, the prediction accuracy for SOH using features from the current cycle was significantly better than using historical data alone.

**Revised:**

1）Methods 1 and 2 provide estimated capacities that are close to the actual capacity when the battery capacity is in a region of slow change. However, when the capacity is in a sharp peak region, Method 1 shows a larger estimation error compared to the actual capacity, whereas Method 2 has a significantly smaller error, which is consistent with the theoretical analysis. Method 3 performs worse than Methods 1 and 2 in regions of slow capacity change due to the interference among the simple combinations of the three features, which negatively impacts the estimation accuracy.

2）When using the first 50% and 70% as the training set, the performance metrics of the three methods ranked from best to worst are: Method 2, Method 1, and Method 3. However, with an increase in the training set size, the performance metrics of all three methods significantly improve, indicating that expanding the training set enhances estimation performance.

**Comment 7**

For battery health estimation, it is recommended to include the following studies to enhance the literature review. “Rapid health estimation of in-service battery packs based on limited labels and domain adaptation” and “Data-Driven Battery State of Health Estimation Based on Random Partial Charging Data”.

**Response:**

Thanks for your comments. We have carefully reviewed and studied the paper you mentioned and have added it to the references.

4. Zhongwei Deng, Le Xu, Hongao Liu, Xiaosong Hu, Bing Wang, Jingjing Zhou,Rapid health estimation of in-service battery packs based on limited labels and domain adaptation,Journal of Energy Chemistry,Volume 89,2024,Pages 345-354,ISSN 2095-4956,https://doi.org/10.1016/j.jechem.2023.10.056.
5. Z. Deng, X. Hu, P. Li, X. Lin and X. Bian, "Data-Driven Battery State of Health Estimation Based on Random Partial Charging Data," in IEEE Transactions on Power Electronics, vol. 37, no. 5, pp. 5021-5031, May 2022, doi: 10.1109/TPEL.2021.3134701.

**Comment 8**

On page 5, the word RUL is not predefined.

**Response:**

Thanks for your comments. We have added the definition of RUL (Remaining Useful Life) in the paper.

Sincerely,

Gengchen Xu
